# Individual, familial and country-level factors associated with oral hygiene practices in children: an international survey

**DOI:** 10.1186/s12903-023-02746-0

**Published:** 2023-01-30

**Authors:** Heba Mohamed Elkhodary, Mohamed Hussein Abdelnabi, Amal Ali Swelem, Heba Jafar Sabbagh, Omar Abd El Sadek El Meligy, Iman Mamdouh Talaat, Enas B. Abdellatif, Yousef Khader, Ola B. Al-Batayneh, Nuraldeen Maher Al-Khanati, Nazik M. Nurelhuda, Sara Alhabli, Mohamed Hassan Mostafa, Shabnum Qureshi, Nafeesa Qureshi, Muhammad Abrar Yousaf, Dunia Taha, Yousef Falah Marafi, Sharifa Nasser Al Harrasi, Sarah Al-Rai, Noha Gomaa, Hala Mattar, Hanin A. Bakhaider, Bahia Samodien, Hanane Lố, Maha El Tantawi

**Affiliations:** 1 Pediatric Dentistry Department, Faculty of Dentistry, King Abdulaziz University, Jeddah, Saudi Arabia; 2 Department of Pedodontics and Oral Health, Faculty of Dental Medicine for Girls, Al Azhar University, Cairo, Egypt; 3 Department of Oral and Maxillofacial Prosthodontics, Faculty of Dentistry, King Abdulaziz University, Jeddah, Saudi Arabia; 4 Department of Removable Prosthodontics, Faculty of Dentistry, Minia University, Minia, Egypt; 5 Department of Prosthodontics, Faculty of Dentistry, Cairo University, Cairo, Egypt; 6 Pediatric Dentistry and Dental Public Health Department, Faculty of Dentistry, Alexandria University, Alexandria, Egypt; 7 Clinical Sciences Department, College of Medicine, University of Sharjah, Sharjah, UAE; 8 Pathology Department, Faculty of Medicine, Alexandria University, Alexandria, Egypt; 9 Department of Public Health, Faculty of Medicine, Jordan University of Science and Technology, Irbid, Jordan; 10 Preventive Dentistry Department, Faculty of Dentistry, Jordan University of Science and Technology, Irbid, Jordan; 11 Department of Oral and Maxillofacial Surgery, Faculty of Dentistry, Syrian Private University, Damascus, Syria; 12 Dental Public Health Division, Faculty of Dentistry, University of Khartoum, Khartoum, Sudan; 13 Department of Education, University of Kashmir, Srinagar, India; 14 NHS Tayside Scotland, Dundee, DD2 2RZ UK; 15 Department of Biology, Faculty of Science and Technology, Virtual University of Pakistan, Lahore, Pakistan; 16 Department of Pediatric Dentistry, Faculty of Dental Medicine, Damascus University, Damascus, Syria; 17 Faculty of Dentistry, Alexandria University, Alexandria, Egypt; 18 Department of Pediatric, Kids Paradise Dental Center, Seeb, Sultanate of Oman; 19 Department of Conservative and Preventive Dentistry, Faculty of Dentistry, Saba University, Sana’a, Yemen; 20 Oral Medicine, Schulich School of Medicine and Dentistry, University of Western Ontario, London, ON Canada; 21 Department of Oral Medicine, Children’s Health Research Institute, London, ON Canada; 22 Western Cape Education Department, Cape Town, South Africa; 23 Department of Pediatric Dentistry, Clinique Dentaire D’Agadir, Agadir, Morocco

**Keywords:** Children, Oral hygiene, Toothbrush, Fluoridated toothpaste, Dental floss, Miswak, Oral health

## Abstract

**Background:**

Maintaining good oral hygiene is key to preventing dental caries and periodontal disease. Children and adolescents with good oral hygiene behaviours are likely to grow into adults with the same behaviours. This study assessed the frequency of using various oral hygiene methods among children and adolescents from different countries and individual, familial and country-level factors associated with the use of these methods.

**Methods:**

A multi-country online survey collected data from caregivers of children in 2020–21 about children’s use of oral hygiene methods including toothbrush, fluoridated toothpaste, mouthwash, dental floss and miswak using self-administered, close-ended questions. Adjusted multilevel logistic regression models were used to assess the relationship between each of the five oral hygiene methods (dependent variables) and the independent factors: sex, age, and history of dental visits (individual factors), mother's education and area of residence (familial factors) as well as country income and region (country-level factors).

**Results:**

A total of 4766 parents/caregivers were included from 20 countries (77.4% Eastern Mediterranean-region and 41.6% lower middle income countries). The most frequent oral hygiene methods were using toothbrush and toothpaste (90% and 60.3%). The use of oral hygiene methods differed by age, sex and history of dental visits as well as mother’s education and area of residence (*P* < 0.05). In addition, children from low income countries had significantly lower odds of using mouthwashes and dental floss than those from high income countries (AOR = 0.55, 95% CI 0.31, 0.98 and AOR = 0.34, 95% CI 0.12, 0.97) whereas children from the European region had higher odds of using mouthwash (AOR = 2.82, 95% CI 1.27, 6.26) and those from the region of the Americas had higher odds of using dental floss (AOR = 3.84, 95% CI 1.28, 11.52) than those from the Eastern Mediterranean region.

**Conclusions:**

The use of various oral hygiene methods is associated with individual, familial and country-level factors. Oral health promotion programs should be developed taking into account these influences.

## Background

Children's oral health is closely associated with their general health and wellbeing. The prevalence of dental caries in school-aged children is very high around the world [1]. When severe, caries can negatively impact children's quality of life, as they would suffer from discomfort, pain, infections, as well as eating and sleeping disorders, which can eventually lead to school absence and learning difficulties [2]. Oral diseases are among the most expensive diseases to treat [3]. In many low-income countries (LICs), the treatment of dental caries in children may surpass the total child healthcare budget [4]. It is, therefore, extremely critical to establish proper oral hygiene behaviors early in life [5].

There are numerous ways to practice oral hygiene. Dental flossing and using a toothbrush with fluoride toothpaste are examples of mechanical procedures [6]. Flossing removes interproximal plaque [7] and it is recommended that parents floss or supervise their children while flossing till the age of 10 [8]. Chemical methods for oral hygiene include plaque removal using mouthwashes on a daily basis combined with brushing [9]. Antimicrobial mouthwashes containing chlorhexidine or herbal mouthwashes are effective and safe in children and can deliver therapeutic ingredients to inaccessible interproximal surfaces [10]. Miswak or chewing sticks represents a traditional method of oral hygiene that is more common in some countries [11]. It has been used for years due to its low cost, availability, and cultural as well as religious reasons [12]. Recent evidence shows that miswak is effective in maintaining OH [13] with antimicrobial, antioxidant, anti-ulcer, and anti-inflammatory effects [14].

Dentists have an important role in improving patients’ oral health by promoting healthy behaviors [15]. Interventions targeting the promotion of oral health behaviors need to respond to the local context, taking into account difference due to availability of supplies, cultures and prevailing norms. Fisher–Owens et al. [16] contend that children’s oral health and health behaviors are affected by multiple interacting influences at the level of the individual, the family and the community. The bulk of dental literature has assessed the impact of individual and familial influence on children’s oral health and oral health behaviors [2, 17, 18] with less understanding of between-country differences and macro-level factors. In addition, available studies have explored between country variations in toothbrushing [19] whereas scarce data is available about the use of other oral hygiene methods. Empirical evidence is needed about the relative role of individual, familial and country level differences in the use of other oral hygiene methods. Such evidence helps develop context-specific health promotion interventions. The present study attempts to fill this knowledge gap by assessing the use of various oral hygiene methods in children and to determine the individual, familial and country-level factors associated with the use of these methods. The null hypothesis of the study was that no differences in the use of oral hygiene methods would be associated with individual, familial or country level factors.

## Methods

### Study design and ethical considerations

This cross-sectional, online survey collected data from caregivers of children in 20 countries (Appendix 1) from August-2020 to February-2021. The study was approved by the Institutional Review Board (IRB) of King Abdulaziz University (# 91-08-20), Public Health Research and Health Statistics-Saudi Center for Disease Control and Prevention (SCDC) (202009091) and the University of Sharjah (REC-20-11-17-01). The study was conducted in full accordance with the Helsinki declaration.

### Participants

Participants were included if they were parents or caregivers of healthy children aged 2–18 years, if they could understand the languages of the survey, and could access the survey through an electronic device and the Internet. Caregivers of medically compromised children were excluded.

Sample size for assessing the frequency of using the different oral hygiene methods was based on hypothesized percentages of using the oral hygiene methods ranging from 5 to 90% with the greatest required sample size obtained at prevalence = 50%. Based on 95% confidence level, 1.5% margin of error and 10% non-response rate, the required sample size was calculated [20] to be 4500 participants. Convenience sampling was used similar to previous studies based on online surveys [21]. Through snowball sampling, study participants were recruited and asked to distribute the survey link to others in their networks.

### The study tool

The questionnaire was based on the World Health Organization Child Oral Health Survey which has been widely used before in its original English version (WHO 2013) as well as in Arabic [22]. The questionnaire consisted of three sections. The first section assessed the child's sociodemographic background including child age in years and sex (male and female), mother’s education (illiterate, elementary or middle school, high school and university or higher) and family residence (urban or rural). The second section assessed the child's oral hygiene practices: the frequency of tooth cleaning, and tooth cleaning methods including toothbrush, fluoridated toothpaste, mouthwash, dental floss and miswak. It also assessed the number of visits the child made to the dentist in the last year (categorized into: one, two, three, four or more, or none). The questionnaire was translated from English to French then back translated again to English. The Arabic used version previously translated and described [23] was also used. The difference between the translated and the original English versions were checked and they were comparable. Content validity (CV) was assessed in the three languages by 10 researchers. The CV index scores were calculated [24] and were found to reflect good content validity (CV index = 0.94, 0.98 and 0.95 respectively).

The questionnaire was uploaded to the electronic survey platform, SurveyMonkey. The settings were managed so that the identity of respondents would be anonymous: no IP addresses were collected, no trackers were installed, and emails or logins were not collected. The survey was preceded by an explanation of the study purpose, and a consent form to be completed before participants could proceed to the survey. After parents/ guardian consented to participate, they were directed to the beginning of the survey. Parents and caregivers were instructed to respond by referring to the youngest child if they were responsible for more than one child. Participants could modify their responses till before submission. Only one submission was allowed per electronic device to prevent duplication. The questionnaire took about 7 min to complete.

### Data collection

The core study team consisted of researchers from King Abdulaziz University, Kingdom of Saudi Arabia and Alexandria University, Egypt. The team invited researchers in their network to collaborate by distributing the questionnaire link to participants in their respective countries. Willing collaborators posted the links on social media inviting parents or guardians fitting the inclusion criteria to respond. The links were posted on groups in FaceBook, Twitter and Instagram as well as to contacts through telegram and WhatsApp. Those receiving the links to the survey were asked to further share it with people in their own networks.

### Statistical analysis

Countries were classified according to the WHO into the following regions: Eastern Mediterranean Region (EMR), African Region (AFR), Western Pacific Region (WPR), European Region (EUR), South-East Asian Region (SEAR) and Region of the Americas (AMR) [25]. Countries were also classified into income levels based on the World Bank classification of gross national income (GNI) using the following cutoff points: low-income countries (LICs), GNI ≤ USD 1035, lower-middle income countries (LMICs), GNI between USD 1036 and 4045, upper middle-income countries (UMICs), GNI between USD 4046 and 12,535 and high-income countries (HICs), GNI ≥ USD 12,536 [26, 27]. Age was categorized into: less than 6 years (preschool age), 6–12 years (schoolchildren age) and 13–18 years (adolescents). There were five dependent variables based on whether the participant used the following oral hygiene methods: toothbrush, fluoridated toothpaste, mouthwash, dental floss and miswak. The independent variables at the individual-level included child’s sex, age groups and dental visits within the last year (categorized by combining any number of visits during the last year together versus none). Familial factors were mother's education and family area of residence. The independent variables also included country-level factors: WHO region and income level. Adjusted multilevel logistic regression models were used to assess the relationship between each of the five dependent variables and the independent factors which were entered as fixed effect factors. Participants were level 1factors clustered in countries which were level 2 factors. In addition, country of residence was entered as random effect variable with robust estimation to address violation of model assumptions. Adjusted odds ratios (AORs) and 95% confidence intervals (CIs) were calculated. IBM SPSS for Windows version 23.0 (IBM Corp., Armonk, NYY, USA) was used for statistical analysis. A *P* value of less than 0.05 was considered statistically significant.

## Results

Table 1 shows the country level and individual characteristics of 4751 parents and caregivers or guardians from 20 countries participating in the study. Most participants were from LMICs (41.6%) and the EMR (77.4%). Males represented 52.3% of the children whose parents and caregivers participated in the study with mean age of 8.5 years, (SD = 4.6). Most children were 6–12 years old (44.3%), with 63.8% reporting that their children had not visited the dentist in the last year. Most mothers were university-educated (68.6%) and 80.7% reported living in urban areas with 44.7% reporting that their children cleaned their teeth once per day while 30.8% cleaned their teeth more than once per day.

**Table 1:** Country-level and individual characteristics of children participating in the study (N = 4751)

Factors		N (%)
Country income level	LICs	592 (12.5)
	LMICs	1976 (41.6)
	UMICs	377 (7.9)
	HICs	1806 (38.0)
WHO region	EMR	3678 (77.4)
	AFR	54 (1.1)
	WPR	106 (2.2)
	EUR	122 (2.6)
	SEAR	592 (12.5)
	AMR	199 (4.2)
Age	Mean (SD)	8.5 (4.6)
	Less than 6 years old	1610 (33.9)
	6–12 years old	2103 (44.3)
	13 years and older	1038 (21.8)
Child’s sex	Male	2484 (52.3)
	Female	2267 (47.7)
Visited the dentist in the last year	Yes	1722 (36.2)
	No	3029 (63.8)
Mother’s education	Illiterate	178 (3.7)
	Elementary/ middle school	418 (8.8)
	High school	898 (18.9)
	University or higher	3257 (68.6)
Area of residence	Urban	3835 (80.7)
	Rural	916 (19.3)
Frequency of tooth-cleaning	Never	184 (3.9)
	More than once per month	179 (3.8)
	Once per week	245 (5.2)
	More than once per week	557 (11.7)
	Once per day	2124 (44.7)
	More than once per day	1462 (30.8)

*LICs* low-income countries, *LMICs* lower middle-income countries, *UMICs* upper middle-income countries, *HICs* high-income countries, *EMR* Eastern Mediterranean Region, *AFR* African Region, *WPR* Western Pacific Region, *EUR* European Region, *SEAR* South-East Asian Region, *AMR* Region of the Americas

Table 2 shows the differences in tooth cleaning methods by country level factors. Most children used toothbrushes (90.0%) and fluoridated toothpaste (60.3%) to clean their teeth and only 12.8% used mouthwashes, 8.5% used dental floss and 5.4% used miswak with statistically significant differences among countries by income level and region (*P* < 0.0001). LMICs had significantly lower percentage of using toothbrush (82.8%) and toothpaste (49.5%) and higher percentage of using miswak (8.4%) than other countries. There was an income level gradient in the use of dental floss with greater percentage reporting use in HICs than UMICs than LMICs than LICs (12.1%, 7.7%, 6.9% vs. 3.2%). Participants in LICs reported the lowest percentage of using mouthwash (7.1%). Table 2 also shows that there was a significantly lower percentage of participants reporting the use of toothbrush and fluoridated toothpaste from SEAR (76.2% and 48.8%) than the other WHO regions whereas a significantly higher percentage of participants from EUR (26.2%) than other regions used mouthwash and a significantly greater percentage reported the use of dental floss in the AMR (31.7%) than in the other regions. Significantly greater percentage of participants reported the use of miswak in AFR (11.1%) and EMR (6.0%) than in the other regions.

**Table 2:** Differences in tooth cleaning methods by country region and income level (n = 4751)

Country-level factors		Toothbrush	Fluoridated toothpaste	Mouthwash	Dental floss	Miswak
N (%)		4278 (90.0)	2865 (60.3)	607 (12.8)	403 (8.5)	256 (5.4)
Income level	LICs	560 (94.6)	371 (62.7)	42 (7.1)	19 (3.2)	28 (4.7)
	LMICs	1637 (82.8)	978 (49.5)	271 (13.7)	136 (6.9)	165 (8.4)
	UMICs	358 (95.0)	262 (69.5)	36 (9.5)	29 (7.7)	10 (2.7)
	HICs	1723 (95.4)	1254 (69.4)	258 (14.3)	219 (12.1)	53 (2.9)
	*P* value	< 0.0001	< 0.0001	< 0.0001	< 0.0001	< 0.0001
WHO region	EMR	3361 (91.4)	2237 (60.8)	418 (11.4)	230 (6.3)	222 (6.0)
	AFR	52 (96.3)	52 (96.3)	7 (13.0)	6 (11.1)	6 (11.1)
	WPR	104 (98.1)	68 (64.2)	21 (19.8)	18 (17.0)	0 (0.0)
	EUR	117 (95.9)	80 (65.9)	32 (26.2)	10 (8.2)	3 (2.5)
	SEAR	451 (76.2)	289 (48.8)	95 (16.0)	76 (12.8)	24 (4.1)
	AMR	193 (97.0)	139 (69.8)	34 (17.1)	63 (31.7)	1 (0.5)
	*P* value	< 0.0001	< 0.0001	0.0001	< 0.0001	< 0.0001

Table 3 shows the individual, familial and country-level factors associated with using various tooth cleaning methods. There were significantly lower odds of using toothbrush in males than females (AOR = 0.69, 95% CI 0.55, 0.83), in children whose mothers were illiterate (AOR = 0.25, 95% CI 0.17, 0.37), had elementary or middle school education (AOR = 0.34, 95% CI 0.24, 0.47) or high school education (AOR = 0.49, 95% CI 0.37, 0.64) than university education. There were significantly higher odds of using toothbrushes among children who were 6–12 year old than 13–18 year old children (AOR = 1.51, 95% CI 1.17, 1.96) and children in urban than rural areas (AOR = 1.40, 95% CI 1.10, 1.79).

**Table 3:** Country-level and individual factors associated with the use of various tooth cleaning methods

Factors		Toothbrush	Toothpaste	Mouthwash	Dental floss	Miswak
Income level	LICs	1.11 (0.34, 3.61)	0.64 (0.27, 1.51)	0.55 (0.31, 0.98)	0.34 (0.12, 0.97)	0.97 (0.19, 4.86)
	LMICs	0.80 (0.27, 2.32)	0.60 (0.29, 1.27)	1.15 (0.71, 1.85)	0.51 (0.21, 1.20)	0.64 (0.13, 3.06)
	UMICs	1.05 (0.26, 4.22)	0.64 (0.24, 1.71)	0.68 (0.34, 1.34)	0.85 (0.27, 2.67)	0.26 (0.03, 2.20)
	HICs	Reference category				
WHO region	EMR	Reference category				
	AFR	2.04 (0.20, 20.87)	22.26 (3.09, 160.61)	1.44 (0.44, 4.69)	1.39 (0.23, 8.21)	10.31 (0.58, 183.25)
	WPR	3.21 (0.52, 19.81)	0.84 (0.32, 2.21)	1.47 (0.74, 2.95)	2.92 (0.97, 8.82)	0 (0, -)
	EUR	1.31 (0.21, 8.34)	0.74 (0.20, 2.68)	2.82 (1.27, 6.26)	1.03 (0.23, 4.63)	0.57 (0.05, 7.12)
	SEAR	0.34 (0.07, 1.72)	0.77 (0.22, 2.69)	1.02 (0.49, 2.13)	2.75 (0.67, 11.23)	0.91 (0.09, 9.43)
	AMR	1.60 (0.35, 7.28)	0.77 (0.28, 2.11)	1.13 (0.58, 2.20)	3.84 (1.28, 11.52)	0.12 (0.01, 1.78)
Age	Less than 6 years old	0.80 (0.61, 1.06)	0.56 (0.47, 0.67)	0.26 (0.20, 0.34)	0.38 (0.28, 0.51)	0.44 (0.30, 0.66)
	6–12 years old	1.51 (1.17, 1.96)	0.80 (0.68, 0.94)	0.50 (0.41, 0.61)	0.54 (0.42, 0.70)	0.47 (0.34, 0.64)
	13- 18 years old	Reference category				
Sex	Male	0.67 (0.55, 0.83)	0.89 (0.79, 1.01)	0.91 (0.77, 1.09)	1.10 (0.89, 1.37)	1.56 (1.18, 2.06)
	Female	Reference category				
Dental Visit in the last year	Yes	0.91 (0.70, 1.19)	0.91 (0.79, 1.05)	1.55 (1.27, 1.89)	1.70 (1.34, 2.15)	0.85 (0.59, 1.24)
	No	Reference category				
Mother’s education	Illiterate	0.25 (0.17, 0.37)	0.41 (0.28, 0.59)	0.53 (0.31, 0.92)	0.52 (0.20, 1.33)	3.33 (1.98, 5.57)
	Elementary/ middle school	0.34 (0.24, 0.47)	0.57 (0.45, 0.73)	0.55 (0.37, 0.80)	0.77 (0.47, 1.26)	1.93 (1.23, 3.03)
	High school	0.49 (0.37, 0.64)	0.59 (0.50, 0.70)	0.81 (0.64, 1.03)	0.81 (0.60, 1.09)	2.00 (1.37, 2.91)
	University or higher	Reference category				
Area of residence	Urban	1.40 (1.10, 1.79)	1.33 (1.14, 1.58)	1.15 (0.89, 1.49)	1.40 (1.00, 1.96)	0.46 (0.33, 0.64)
	Rural	Reference category				

Using fluoridated toothpaste was not significantly associated with country income but was significantly higher in AFR than EMR although the estimate was not precise as indicated by the very wide CI (AOR = 22.26, 95% CI 3.09, 160.61). It was significantly higher in children in urban than rural areas (AOR = 1.33, 95% CI 1.14, 1.58). There were lower odds of using toothpaste among children whose mothers were illiterate (AOR = 0.41, 95% CI 0.28, 0.59), had elementary or middle school education (AOR = 0.57, 95% CI 0.45, 0.73) or high school education (AOR = 0.59, 95% CI 0.50, 0.70) than university educated mothers. Children less than 6 years old (AOR = 0.56, 95%CI%: 0.47, 0.67) and 6–12 years old (AOR = 0.80, 95% CI 0.68, 0.94) had lower odds of using fluoridated toothpaste than 13–18 year old children.

Using mouthwash was significantly lower among children in LICs than HICs (AOR = 0.55, 95% CI 0.31, 0.98) and higher in EUR (AOR = 2.82, 95% CI 1.27, 6.26) than EMR and in children visiting the dentist last year (AOR = 1.56, 95% CI 1.28, 1.90). There were significantly lower odds of using mouthwash among children whose mothers were illiterate (AOR = 0.53, 95% CI 0.31, 0.92), had elementary or middle school education (AOR = 0.55, 95% CI 0.37, 0.80) than university educated mothers as well as among children younger than 6 years old (AOR = 0.26, 95% CI 0.20, 0.34) and 6–12 year old children (AOR = 0.50, 95% CI 0.41, 0.61).

There were significantly lower odds of using dental floss in LICs than HICs (AOR = 0.34, 95% CI 0.12, 0.97), in younger than 6 years (AOR = 0.38, 95% CI 0.28, 0.51) and 6–12 year old (AOR = 0.54, 95% CI 0.42, 0.70) than 13–18 year old children. There were significantly higher odds of using dental floss among children in AMR (AOR = 3.84, 95% CI 1.28, 11.52) than EMR, and children who visited the dentist last year (AOR: 1.70, 95% CI 1.34, 2.15).

The odds of using miswak were significantly higher among males than females (AOR = 1.56, 95% CI 1.18, 2.06), children whose mothers were illiterate (AOR = 3.33, 95% CI 1.98, 5.57), with elementary or middle school education (AOR = 1.93, 95% CI 1.23, 3.03) or high school education (AOR = 2.00, 95% CI 1.37, 2.91) than university education. There were significantly lower odds among children younger than 6 years (AOR = 0.44, 95% CI 0.30, 0.66) and 6–12 year old (AOR = 0.47, 95% CI 0.34, 0.64) than 13–18 year old and children in urban than rural areas (AOR = 0.46, 95% CI 0.33, 0.64).

## Discussion

In the present study, most children used toothbrush and to a lesser extent fluoridated toothpaste whereas mouthwash, dental floss and miswak were far less common. In addition to the individual variations by sex, age and history of dental visits in the last year and familial factors such as mothers’ education and area of residence that were previously reported in the literature, the study suggests variations by country region and country income indicating possible cultural, healthcare systems and contextual influences on oral hygiene practices in children. The study hypothesis is thus partially supported.

The present study had some limitations. First, data were collected electronically, and this introduced some selection bias where participants were likely to be highly educated. The convenience sample may, thus, not be representative of the general population in different countries. However, this data collection method and sampling strategy have been previously used in studies conducted during the pandemic due to restricted mobility and physical distancing [28–30]. Second the study used a cross-sectional design that does not prove causality but only suggests associations. Thus, for example, the higher odds of using dental floss associated with history of dental visit may indicate that encounters with a dentist promote flossing or, alternatively, that those who floss are more likely to visit the dentist. Third, the sample included unequal number of participants from different countries and regions. However, we accounted for between-country variations by using multi-level modelling with robust estimation to address violations of model assumptions. The study fills a knowledge gap by providing evidence about the frequency of using various oral hygiene methods in children and adds to the existing literature which mostly reports on the use of toothbrush and toothpaste [19]. The study also included participants from various countries which increases the generalizability of findings in addition to addressing individual-and country-level factors.

The study had several important findings. Individual and familial factors were significantly associated with the use of most oral hygiene methods: males had significantly lower odds than females of using toothbrushes, in agreement with the literature [19, 31] which may be because females place greater importance on their oral health than males [32]. The exception to this association was that males had higher odds of using miswak which needs to be explored in future studies. Similar to previous studies, we observed an association between the use of oral hygiene methods and history of dental visits [33]. However, in this study, dental visits were associated with using mouthwash and dental floss, showing dentists' role in educating patients about their use. This association was not observed in case of toothbrush and toothpaste indicating their more widespread use and that they may be considered as part of personal grooming habits not exclusively dependent on dentists’ advice. The present study also showed urban–rural differences in preferred oral hygiene methods with greater odds of using toothpaste and toothbrush in urban areas similar to previous studies [34–36]. Also, having less educated mothers was associated with no use of toothbrushes, toothpaste and mouthwash similar to previous studies [31, 37]. The exception was children of illiterate mothers who had higher odds of using miswak which, together with its use in rural areas, may be attributed to its lower cost compared to the other oral hygiene methods. The study also showed that preschool and school children were less likely to use toothpaste, mouthwash, floss and miswak than adolescents. This may be party attributed to adolescents assuming the responsibility of their own oral hygiene as they grow and partly following advice not to use mouthwash in younger ages to avoid harm due to accidental swallowing or because floss cannot be effectively used by young children on their own. Pending confirmation at country level, this finding may have implications for school-based health education programs to promote the use of toothpaste especially in elementary schools.

The study also showed that the use of oral hygiene methods was associated with country-level factors. The use of toothbrush and fluoridated toothpaste did not differ significantly between lower income countries and HICs which agrees with the dental literature [19, 38]. However, the current study showed that children from LICs had significantly lower odds of using mouthwashes and dental floss than children from HICs. This may be explained by the greater purchasing power in HICs. Children in LICs, on the other hand, usually have limited ability to buy these products [19]. In addition, oral health promotion campaigns encouraging the use of oral hygiene methods were reported to be available in most HICs but are less frequent in LICs [38, 39].

The present study also showed differences in the use of oral hygiene methods by WHO region. Children from the EUR and AMR regions had significantly higher odds of using mouthwashes and dental floss than children from EMR. This could be due to the greater accessibility of these oral hygiene products in these regions [19] in addition to cultural and healthcare system contextual factors although limited evidence is available to explain these differences and further studies are needed for more in depth understanding. Bivariate analysis shows higher percentage of using miswak among children in AFR and EMR than all other regions with no significant difference observed between the two regions in the multivariable analysis when all other factors were taken into consideration. Miswak is more popular in Muslim countries and some countries in Africa and the Middle East and in low socioeconomic areas [36, 40]. The inclusion of this traditional method in studies assessing oral hygiene behaviours may shed light on already existing behaviours instead of ignoring them and dedicated resources to replace them with the practices that are recommended by dental specialists based on evidence derived from other parts of the world.

## Conclusions

Toothbrush and fluoridated toothpaste were the most frequently used oral hygiene methods in children from 20 countries. Differences were observed among children and adolescents by country region and income level even after accounting for individual and familial factor. Mouthwash and dental floss were less prevalent in LICs and more prevalent in EUR and AMR respectively whereas miswak was more prevalent in EMR and AFR. Oral health initiatives promoting positive oral hygiene behaviours should be tailored to contextual effects that differ among countries and adapted to local needs and demands. It is important to design these initiatives using the principles of participatory action research to account for the voices and input of the local communities.

## Data Availability

Data used for this study are available from the first author upon reasonable request. Data are not posted in open access data servers because they will be used for further publications.

## Appendix 1

**Table A:** Number of participants by country

Countries	Number of participants	%
Australia	50	1.0
Canada	126	2.6
Egypt	659	13.8
India	592	12.4
Iraq	108	2.3
Jordan	218	4.6
Kuwait	435	9.1
Morocco	77	1.6
Oman	197	4.1
Pakistan	504	10.6
Palestine	92	1.9
Philippines	56	1.2
Saudi Arabia	714	15.0
South Africa	54	1.1
Sudan	249	5.2
Syria	203	4.3
United Arab Emirates	92	1.9
United Kingdom	123	2.6
United States	73	1.5
Yemen	144	3.0
Total	4766	100

## Abbreviations

IRB: Institutional Review Board
SCDC: Saudi Center for Disease Control and Prevention
WHO: World Health Organization
CV: Content validity
EMR: Eastern-Mediterranean-region
AFR: African Region
WPR: Western Pacific Region
EUR: European Region
SEAR: South-East Asian Region
AMR: Region of the Americas
GNI: Gross national income
LIC: Low-income countries
LMIC: Lower-middle-income-countries
UMIC: Upper middle-income countries
HIC: High-income countries
AOR: Adjusted odds ratios
CIs: Confidence intervals
SPSS: Statistical Package for Social Sciences
